# Skeleton-Based Action Quality Assessment with Anomaly-Aware DTW Optimization for Intelligent Sports Education

**DOI:** 10.3390/s25237160

**Published:** 2025-11-24

**Authors:** Wen Fu, Wenze Fang, Jiahao Huang, Kexin Zhu, Renguang Chen, Chen Feng

**Affiliations:** 1 College of Sports Science, Fujian Normal University, Fuzhou 350100, China; fuwen@fjnu.edu.cn; 2 College of General Education, Beijing Normal Hong Kong Baptist University, Zhuhai 519087, China; wenzefang@uic.edu.cn; 3 College of Computer and Cyber Security, Fujian Normal University, Fuzhou 350100, China; 4 Department of Computer Science, Taiwan Sun Yat-sen University, Taiwan 510275, China; 5 School of Information Engineering, Fuzhou Polytechnic, Fuzhou 350108, China

**Keywords:** pose estimation, action quality assessment, skeleton-based human anomaly detection, dynamic time warping, intelligent physical education

## Abstract

In intelligent sports education, current action quality assessment (AQA) methods face significant limitations: regression-based methods are heavily dependent on high-quality annotated data, while unsupervised methods lack sufficient accuracy and degrade performance when handling long-duration sequences. To address these challenges, this paper introduces a novel indirect scoring method integrating action anomaly detection with a Quick Action Quality Assessment (QAQA) algorithm. In this method, the proposed anomaly detection module dynamically adjusts action quality scores by identifying and analyzing acceleration outliers between frames, effectively improving the robustness and accuracy of sports AQA. Moreover, the QAQA algorithm utilizes a multi-resolution approach, including coarsening, projection, and refinement, to significantly reduce computational complexity to O(n), alleviating the computational burden typically associated with long sequence analyses. Experimental results demonstrate that our method outperforms traditional methods in execution efficiency and scoring accuracy. The proposed system improves algorithmic performance and effectively contributes to intelligent sports training and education.

## 1. Introduction

The societal shift towards healthier lifestyles has spurred a significant rise in the popularity of mind-body exercises. However, improper execution of these exercises frequently diminishes training efficacy and may even lead to physical injuries. This critical issue has garnered considerable research attention [1,2].

Action quality assessment (AQA) is a critical challenge in traditional Chinese Qigong (e.g., Baduanjin, Tai Chi), a mind-body discipline valued for its rich cultural heritage and documented health benefits [3,4,5]. However, its pedagogy is fundamentally limited, relying on subjective instructor feedback rather than quantitative metrics and real-time correction. This traditional approach severely hinders the standardized instruction and scientific dissemination of Qigong.

AI-driven systems offer a compelling solution to these pedagogical limitations. Underpinned by recent advances in computer vision, they can provide objective AQA and real-time corrective feedback. This capability, leveraging deep learning for fine-grained pose and trajectory analysis, enables a robust, quantitative evaluation of action quality that transcends traditional subjective methods, thereby enhancing training efficacy and facilitating standardized dissemination.

Current mainstream methods for AQA fall into two main categories: supervised and unsupervised learning. Supervised frameworks [6,7,8,9,10] mainly use end-to-end deep learning architectures, employing spatio-temporal convolutions or recurrent neural networks to map video sequences to quality scores directly. Although these methods achieve high precision for specific action scoring tasks, they have three inherent limitations. First, they overlook the rich spatio-temporal information present in human skeleton data. Second, uniform sampling strategies create problems with action phase segmentation, resulting in the loss of critical features during key movement stages. Third, these models heavily depend on fine-grained annotations of the datasets, posing the dual challenges of limited domain adaptability and high annotation costs.

To overcome these annotation-related limitations, researchers have explored unsupervised methods based on Dynamic Time Warping (DTW) [2,11,12,13,14]. These methods construct evaluation metrics based on similarity between movements, calculating the nonlinear alignment distance between test sequences and reference templates. Compared to supervised methods, the DTW approach reduces the need for data annotation and enhances the model’s sensitivity to temporal variations in actions. However, practical applications have exposed two significant drawbacks of DTW-based methods. Firstly, distance metrics are sensitive to noise, leading to possible inaccuracies in assessment. Secondly, the computational complexity of DTW algorithms grows exponentially when handling complex, long-duration actions. This exponential complexity results in low efficiency, making it unsuitable for real-time applications.

In response to the issues described above, this paper proposes a fast AQA method based on anomaly detection optimization and indirect scoring. First, an anomaly detection module is developed to address the problem of outliers and noise present in outdoor action sequences. This module identifies abnormal states of keypoints in the temporal dimension and calculates score adjustments according to specific situations. Second, we build a fast action scoring algorithm that efficiently computes the final scores of sports actions. This algorithm simplifies the dynamic programming score matrix using coarsening, projection, and refinement techniques. Then, it integrates the adjustment strategies calculated by the anomaly detection module to determine the final scores. Finally, we developed a new video dataset that features traditional Chinese Qigong movements and validated the effectiveness and practicality of our proposed method.

The main contributions of this paper can be summarized as follows.

- We propose Quick Action Quality Assessment (QAQA), a fast AQA algorithm based on ACDTW, which significantly reduces the algorithm time complexity to O(n). This improvement addresses computational efficiency problems in the assessment of long-duration action sequences, improving the algorithm’s performance while maintaining high assessment accuracy. Furthermore, the method shows strong generalization capability for real-world applications in Qigong teaching evaluation.
- We introduce an action anomaly detection module based on the DBSCAN clustering algorithm. This module dynamically adjusts the threshold parameters in the action matching algorithm by comparing differences in the acceleration outlier counts between standard and test actions. This strategy improves the model’s ability to detect and differentiate abnormal actions, thereby boosting the accuracy of action assessments.
- We build a novel dataset explicitly designed for AQA in traditional Chinese Qigong. This dataset includes two Qigong routines (Baduanjin and Yijinjing) comprising 22 subactions. Ablation and comparative experiments demonstrate the effectiveness and superiority of our proposed method on this dataset.

## 2. Related Work

### 2.1. Human Pose Estimation

Human Pose Estimation, which extracts skeletal keypoints for tasks like Action Quality Assessment (AQA), is dominated by two paradigms: top-down methods [15,16], whose performance is bottlenecked by the initial person detection, and more efficient bottom-up approaches [17] that contend with a complex keypoint association stage. The modern research landscape is driven by the accuracy–efficiency trade-off, leading to the integration of lightweight backbones, attention, and Transformers [16,18,19,20], culminating in robust real-time models like RTMPose, developed within the comprehensive MMPose toolbox [21,22]. However, the performance of these purely vision-based systems degrades under challenging conditions like occlusion. Consequently, to enhance robustness, a complementary line of research augments visual data with explicit kinematic modeling, using features like motion trajectories and joint angles for more reliable AQA [23,24].

### 2.2. Action Quality Assessment

Supervised AQA, while effective, is fundamentally hampered by its reliance on extensive, task-specific annotations, which restricts model generalizability [6,25,26]. Although more sophisticated architectures and multi-modal fusion techniques have advanced performance, they do not alleviate this core dependency and often introduce significant model complexity [6,7,8,9,10]. Consequently, a significant research thrust has focused on reducing or eliminating this label dependency. Semi-supervised learning (SSL) accomplishes this by leveraging unlabeled data, either through pseudo-labeling with teacher-student frameworks [27,28,29] or by imposing temporal consistency constraints [30]. Pushing this further, unsupervised learning (USL) methods operate without any annotations, typically by reframing AQA as a metric-based comparison task [31] or by learning robust feature representations directly from unlabeled skeletal data [32,33].

### 2.3. Dynamic Time Warping Algorithm

DTW is a foundational algorithm for comparing time series of varying lengths, first proposed by Sakoe et al. [34] and widely applied in fields like speech recognition [3,35,36]. In AQA, its capacity for non-linear sequence alignment is crucial for template-based methods [11,12,13], enabling diverse applications from clinical movement analysis [1,14] to detailed skill scoring and feedback generation [2,37].

Despite its utility, canonical DTW suffers from significant limitations. Its quadratic computational complexity poses a challenge for long sequences, while its tendency to produce singular alignments can lead to inaccurate results. Consequently, a line of research has focused on optimization. To improve efficiency, algorithms like FastDTW [38] and SparseDTW [39] were developed to reduce complexity. To enhance alignment accuracy, methods such as Derivative DTW [40] were designed to align sequence shapes, while ACDTW [41] employs adaptive penalties to resolve singularity issues.

## 3. Methods

**Figure 1:**
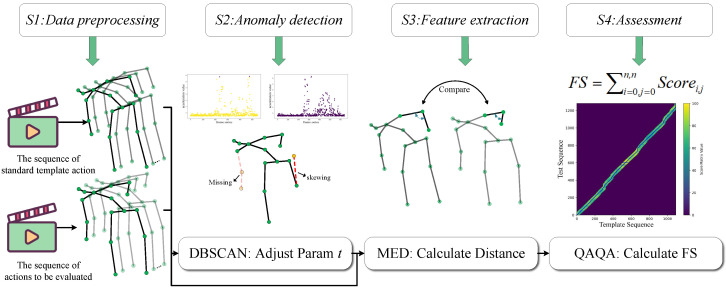
The overview of our annotation-free scoring framework. It first extracts 3D skeletal keypoints from both standard and evaluation videos, which are used for two parallel purposes: to generate kinematic feature sequences for comparison, and to compute an anomaly adjustment parameter *t*, based on acceleration (The purple and yellow points denote two instances from the acceleration clustering results of the anomaly detection module.). The final quality assessment is then determined by our QAQA algorithm, which compares the feature sequences and leverages *t* to modulate the score based on detected irregularities.

Existing AQA methods are hindered by a dependency on large, annotated datasets and a failure to handle unsegmented real-world videos, which limits their generalizability and practical utility. To overcome these barriers, we propose an unsupervised, skeleton-based AQA framework. As depicted in Figure 1, our annotation-free pipeline consists of four sequential stages: (S1) data extraction and processing, (S2) anomaly detection, (S3) action feature construction, and (S4) final score computation via our proposed QAQA algorithm. This design ensures broad applicability across diverse exercise scenarios without requiring model retraining.

### 3.1. S1: Data Extraction and Processing

In human AQA, mainstream pose estimation algorithms typically represent action characteristics using 17 skeletal keypoints. These algorithms are based on simplified biomechanical models and validated through engineering practices. Among these algorithms, MMPOSE is widely adopted for keypoint extraction due to its flexibility and scalability [42,43,44]. Thus, in this study, we use MMPOSE to extract 3D coordinates of human skeletal keypoints from exercise videos. The processes are described in detail below.

**Figure 2:**
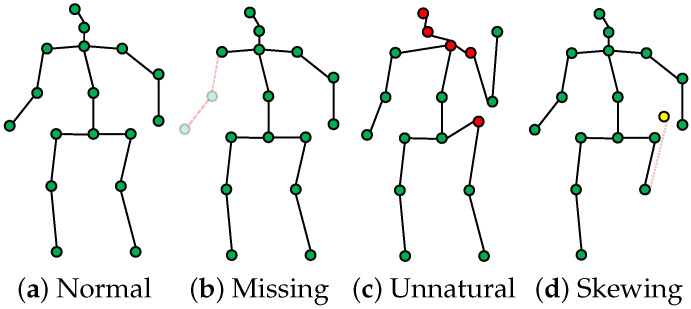
Anomalous keypoints during data preprocessing, these raw keypoints are often corrupted by artifacts, including partial occlusions (missing keypoints), anatomically implausible poses that violate biomechanical constraints (unnatural), and high-frequency jitter or drift (skewing).

The initial stage of our framework is to extract 3D skeletal keypoints from the input videos. To this end, we adopt a multi-stage pipeline leveraging the MMPose toolbox [42,43,44]. Initially, the RTMDet model [45] identifies human bounding boxes. Subsequently, a top-down estimator with a ResNetV1D50 backbone [46] extracts 2D keypoints within these boxes, which are then lifted to 3D coordinates using VideoPose3D [47]. The final output for each video is a time-series of 17 standard 3D skeletal keypoints (e.g., head, shoulders, hips, limbs), providing a complete spatial representation of the pose in each frame, as visualized in Figure 2a.

### 3.2. S2: Anomaly Detection

In outdoor sports training, the accuracy of human keypoint detection can be significantly affected by factors such as camera angles, lighting conditions, object occlusions, and algorithmic errors. Subsequently, these issues affect the assessment of sports actions. Figure 2 illustrates several common anomalies, including missing keypoints Figure 2b, abnormal shifts Figure 2d, and unnatural pose keypoints Figure 2c.

To address these challenges and improve the accuracy of action assessment, we propose a pose feature enhancement method based on anomaly detection. This method identifies anomalies introduced during data preprocessing and dynamically adjusts subsequent scoring based on the type and severity of detected anomalies.

The processing flow of the anomaly detection module consists of three primary steps: First, extract the acceleration sequence for both the template and the test keypoint data. Second, determine the DBSCAN parameters (ϵ) and detect outliers in the acceleration sequences. Finally, calculate the threshold coefficient *t* based on the outliers detected in Step 2.

Step 1 (Extracting Acceleration Sequences): Traditional Chinese Qigong actions typically involve slow stretching, twisting, and balancing movements, leading to relatively stable changes in the skeletal keypoints. To improve computational efficiency, we remove data from the spine, chest, and hip midpoint keypoints, as their accelerations show minimal variation. Thus, we retain acceleration data from 14 keypoints.

Each keypoint sequence is approximated as a uniform motion by calculating Euclidean distance differences of every two frames in the 3D space. The acceleration for keypoint KP in frame *i* is calculated using Equation (1):

(1) $$\begin{array}{cccc} & & acceleration\left(KP,i\right)=\frac{ed(KP_{i+2}-KP_{i})}{time^{2}} & \end{array}$$

Here, KP represents the 3D coordinates of a specific keypoint, i∈[1,…,n−2], *n* is the total frame count, ed denotes the Euclidean distance function, and time is the interval between two consecutive frames.

Step 2 (Outlier detection with DBSCAN): We employ the DBSCAN [48] for anomaly detection within individual keypoint acceleration series due to its unsupervised nature, its ability to identify outliers without pre-specifying the number of clusters, and its proven efficacy in density-based outlier detection. Since the motion ranges of the keypoints vary significantly across different actions, using a fixed neighborhood radius could result in suboptimal clustering. Thus, we dynamically determine the DBSCAN parameter ϵ using a *k*-distance method. The *k*-distance is the distance from each acceleration data point to its *k*-th nearest neighbor, computed by Equation (2):

(2) $$\begin{array}{cccc} & & dists=KDist(acc,minSample) & \end{array}$$

Here, minSample denotes the minimum number of samples required to form a cluster, and acc denotes the acceleration sequence calculated by Equation (1).

We then select ϵ as the point with the maximum slope change in the sorted *k*-distance graph, as shown in Equation (3):

(3) $$\begin{array}{cccc} & & selectEpsilon\left(dists\right)=\max\left(\frac{dists_{j+1}-dists_{j}}{j+1-j}\right) & \end{array}$$

**Figure 3:**
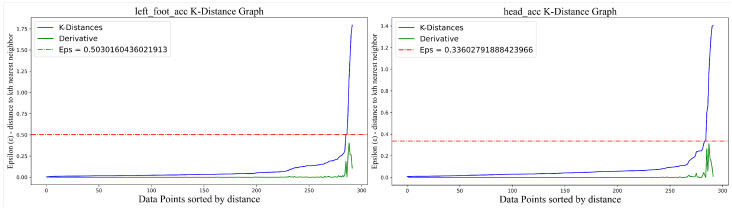
Dynamic determination of the optimal DBSCAN parameter ϵ via the maximum slope of the k-distance curve.

Here, dists represents the sorted *k*-distance values, j∈[1,n−2] indexes distances. Equation (3) identifies the point with the maximum slope difference, which sets the value of the parameter ϵ. The selection of ϵ is illustrated in Figure 3.

Figure 3 visualizes the *k*-distance graph. The vertical axis shows the maximum distance from each data point to its nearest neighbor required for the minimum cluster size, while the horizontal axis indicates the index of data points sorted by distance. The green curve represents the derivative of the *k*-distance values, and the red dashed line marks the optimal position of the parameter ϵ. The DBSCAN algorithm selects the vertical coordinate value at this position as its parameter ϵ. This approach ensures accurate clustering for each keypoint under varying conditions, improving the robustness of anomaly detection.

Therefore, given the input action sequences, we denote the total number of acceleration outliers in the standard action sequence as $o_{1}$ and in the test video sequence as $o_{2}$. By entering the determined parameters (ϵ, minSample) and the acceleration sequences into the DBSCAN model, we can calculate $o_{1}$ and $o_{2}$. The calculation method for counting outliers is shown in Equation (4):

(4) $$\begin{array}{cccc} & & countOutliers(DBSCAN(acc,minSamples,\epsilon )) & \end{array}$$

Here, countOutliers is a function that returns the number of detected outliers, and DBSCAN refers to the DBSCAN clustering model.

Step 3 (Threshold Coefficient Calculation): We calculate the threshold coefficient *t* based on the ratio of the absolute difference between the number of outliers in the standard action sequence ($o_{1}$) and the test action sequence ($o_{2}$), given by $\frac{|o_{1}-o_{2}|}{o_{1}}$. The specific threshold values assigned according to this ratio are detailed in Equation (5).

(5) $$\begin{array}{cccc} & & setThreshold\left(o1,o2\right)=\left\{\begin{array}{cc} 1, & \frac{\left|o1-o2\right|}{o1}=0\\ 0.2, & 0<\frac{\left|o1-o2\right|}{o1}\leq \;0.3\\ 0.15, & 0.3<\frac{\left|o1-o2\right|}{o1}\leq \;0.5\\ 0.1, & 0.5<\frac{\left|o1-o2\right|}{o1} \end{array}\right. & \end{array}$$

It is important to note that some discrepancies between standard and test actions are expected. However, if both actions have identical distributions of joint acceleration outliers (i.e., $\frac{|o_{1}-o_{2}|}{o_{1}}=0$), it strongly suggests the possibility of cheating. To address this, assignments suspected of cheating are directly assigned a threshold coefficient of 1, indicating that no further scoring is required.

The detailed role and usage of this parameter *t* will be described further in Section 3.4. The complete anomaly detection process is summarized in Algorithm 1:
**Algorithm 1:** Human Skeleton Keypoint Anomaly Detection
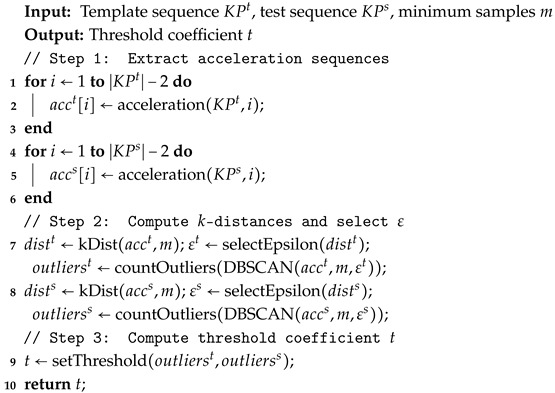


### 3.3. S3: Construction of Action Feature

This study integrates motion state features, including limb angles, body orientation, and shoulder-hip angles, to construct a complete 3D human motion representation. The coordinate system is reconstructed with the neck as the origin based on the 3D spatial coordinates of 17 skeletal keypoints. Subsequently, the limb angles (covering both upper and lower limb joints) and body orientation features are calculated in both 2D and 3D space.

To address the morphological characteristics of Qigong action, we select high-attention skeletal keypoints and establish an 11-dimensional angle feature set, as detailed in Table 1.

The keypoints are labeled using a number-name format, with their mathematical representation given in Equation (6):

(6) $$\begin{array}{cccc} & & \theta =\arccos\left(\frac{\vec{AB}\;\cdot \;\vec{CD}}{|\vec{AB}\left|\times \right|\vec{CD}|}\right) & \end{array}$$

Here, $\vec{AB}$ and $\vec{CD}$ represent vectors between two joint nodes, and θ is the angle between these vectors. For simplicity, skeletal keypoints are abbreviated as KP.

For spatial limb segment features, we define four proximal limb segments based on anatomical landmarks: left arm (KP 8, 14, 15, and 16), right arm (KP 8, 11, 12, and 13), left leg (KP 0, 1, 2, and 3), and right leg (KP 0, 4, 5, and 6). By calculating the spatial angles between the center of each limb segment and a reference point at the hip (using the geometric center calculation in Equation (7)), we establish four-dimensional kinematic parameters for limb movement:

(7) $$\begin{array}{cccc} & & p=\left(\frac{\sum x_{i}}{4},\frac{\sum y_{i}}{4},\frac{\sum z_{i}}{4}\right),i\in \left[1,4\right] & \end{array}$$

In Equation (7), $x_{i},y_{i}$, and $z_{i}$ denote the 3D spatial coordinates of the selected limb block, and $p_{0}$ represents the 3D coordinates of the center point.

The angle between the center point of a limb block and the midpoint of the spine is then calculated as follows:

(8) $$\begin{array}{cccc} & & \alpha =\arccos\left(\frac{\vec{M}\;\cdot \;\vec{op_{0}}}{|\vec{M}\left|\times \right|\vec{op_{0}}|}\right) & \end{array}$$

Here, $\vec{M}$ denotes the spine vector, $\vec{op_{0}}$ is the vector from the neck to the center of the limb block, and α is the angle between these two vectors. This calculation yields four spatial upper and lower limb block features, providing important references for subsequent AQA.

To obtain the angle between the coordinates of the center point and the midpoint of the spine, four spatial upper and lower limb block features can be obtained using Equation (8). These features provide important references for subsequent AQA. $\vec{M}$ represents the spine vector, $\vec{op_{0}}$ represents the vector from the neck to the center point of the limb block, and α represents the angle between the center point vector of the limb block and the spine vector.

We construct a hierarchical orientation system to represent human body orientation. Using vectors connecting the shoulders (KP-11, 12) and hips (KP-0, 1), we establish a three-dimensional anatomical reference in space. The vector projection is then used to convert discrete directional parameters into continuous radian values, setting the forward direction as 1.5π and the reverse direction as 0.5π. These are denoted as orientation features $\beta_{1}$ and $\beta_{2}$.

When calculating the opening angles between both hands and both feet, these features are not originally radian values, so we normalize them to radians. Specifically, if the distance between the left and right feet is greater than 0.5 times the distance between the left and right shoulders, the opening angle of the feet is set to 1.5π. Otherwise, it is set to 0.5π. Similarly, if the distance between the left and right wrists is greater than 1.5 times the distance between the left and right shoulders, the opening angle of the hands is set to 1.5π; otherwise, it is set to 0.5π. These two features are denoted as $\beta_{3}$ and $\beta_{4}$.

For four groups of contralateral limb combinations, such as the left upper limb–left lower limb (KP-15, 16, 2, 3) and the right upper limb–right lower limb (KP-12, 13, 5, 6), sagittal plane movement angles are calculated using Equation (6) and denoted as $\gamma_{1},\gamma_{2},\gamma_{3},\gamma_{4}$.

Finally, by combining limb angles, body orientation, and shoulder-hip joint angles, we obtain a total of 27-dimensional limb motion angle features: *F* = [$\theta_{1}$ … $\theta_{1}$, $P_{1}$ … $P_{4}$, $\alpha_{1}$ … $\alpha_{4}$, $\beta_{1}$ … $\beta_{4}$, $\gamma_{1}$ … $\gamma_{4}$]. These features provide comprehensive input data for subsequent action similarity calculations and action scoring methods. To further optimize the efficiency of AQA, we designed a fast action quality scoring algorithm QAQA to address performance bottlenecks in long-sequence calculations.

### 3.4. S4: Quick Action Quality Assessment

To optimize the time complexity of the ACDTW algorithm [41] for the computation of action similarity, we propose the QAQA algorithm, as detailed in Algorithm 2.

QAQA uses an approximate path approach that incrementally refines the final alignment by coarsening, projecting, and refining the dynamic programming score matrix.

**Figure 4:**
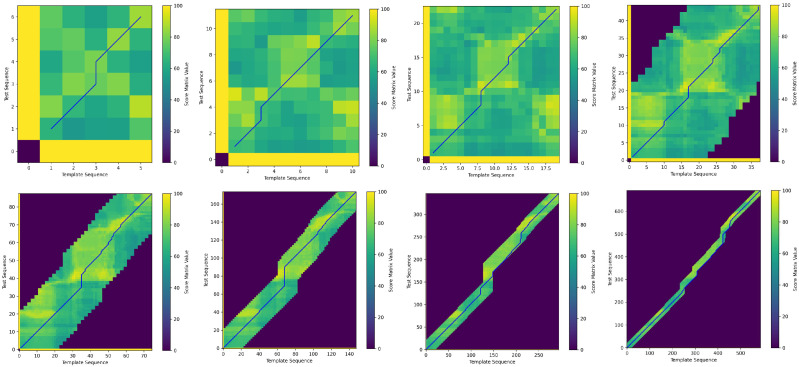
QAQA iterative process from low resolution to high resolution.

Figure 4 illustrates the iterative process of QAQA, where blue lines indicate backtracking paths from low to high resolution, and darker regions represent areas with uncomputed scores.
**Algorithm 2:** Quick Action Quality Assessment (QAQA)
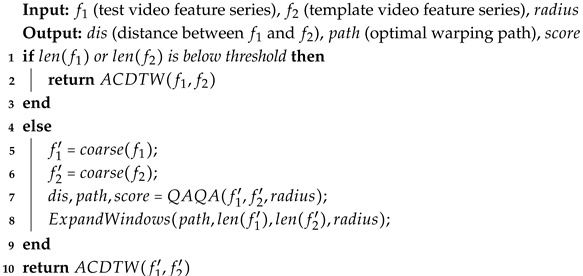


In the coarsening step, QAQA first performs coarse-grained processing of the two time series. According to Equation (9), the series are downsampled by averaging pairs of adjacent features, reducing them to a predefined minimum length.

(9) $$\begin{array}{cccc} & & \mathrm{coarse}\left(F\right)=\frac{F_{i+1}-F_{i}}{2},i\in \left\{1,3,5,\ldots ,n-1\right\} & \end{array}$$

Using the downsampled series, the dynamic programming distance matrix and action score matrix are computed using ACDTW with a penalty function, resulting in a coarse-grained backtracking path. A weighting factor, typically based on local features or time series attributes, adjusts the match cost for each pair of matching points. The calculation methods for ACDTW are shown in Equations (10) and (11).

(10) $$\begin{array}{cccc} & & C\left(x_{i-1,j}\right)=\frac{2\times \max(a,b)}{a+b},i\in \left\{1,3,5,\ldots ,n-1\right\} & \end{array}$$

(11) $$\begin{array}{c} ACDTW\left(i,j\right)=MED\left(i,j\right)+\min\left\{\begin{array}{c} ACDTW(i-1,j-1)\\ ACDTW\left(i-1,j\right)+C\left(x_{i-1,j}\right)\times MED\left(i,j\right)\\ ACDTW\left(i,j-1\right)+C\left(x_{i,j-1}\right)\times MED\left(i,j\right) \end{array}\right. \end{array}$$

Equation (10) defines the penalty function, where *N* is the number of times each time series point is matched, and *a* and *b* are the lengths of the two series. Equation (11) describes the ACDTW computation, where *i* and *j* index the frames in the standard and test action sequences, respectively.

During the projection step, the algorithm calculates the extended window range based on the coarse-grained backtracking path, the radius parameter, and the two feature sequences. This window limits the computational region. Details of the extended window algorithm are provided in Algorithm 3.
**Algorithm 3:** ExpandWindows
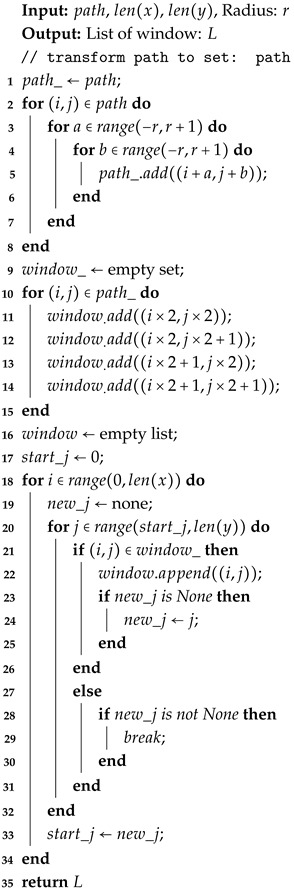


In the refinement step, the two time series are upsampled, and ACDTW updates the dynamic programming distances only within the specified window. Based on these distances, the backtracking path is recalculated. This cycle of coarsening, window computation, and refinement repeats until the full-resolution distance matrix and the final backtracking path are obtained, at which point the iteration terminates.

Finally, the action score is calculated along the full-resolution backtracking path using Equations (12)–(14). For each frame along the backtracking path, the action score is computed according to Equation (13). Based on the threshold coefficient *t* (determined by Equation (5) in Section 3.2), different penalty levels are applied to the action similarity distances. The frame-level scores are then summed and averaged to yield the final score.

(12) $$\begin{array}{cccc} & & q=\frac{|F_{test}-F_{tem\;p}|}{F_{test}} & \end{array}$$

(13) $$\begin{array}{cccc} & & S_{ij}=\left\{\begin{array}{cc} S_{ij}, & q\leq \;t\\ S_{ij}\times \left(1-q+t\right), & t<q\leq \;1\\ 0, & q>1\vee t=1 \end{array}\right. & \end{array}$$

(14) $$\begin{array}{cccc} & & score=\frac{\sum_{i,j}s_{i,j}}{n} & \end{array}$$

In Equation (12), $F_{test}$ and $F_{tem\;p}$ denote the features of the test and standard action sequences at frame *i*, respectively, with *q* measuring the discrepancy between the evaluated and standard actions. In Equation (13), $S_{ij}$ is the score of frame *i* along the backtracking path. If t=1, the score is set to 0 for that frame. In Equation (14), *n* is the total number of points along the backtracking path.

### 3.5. Unsupervised Labeled Qigong Datasets

**Figure 5:**
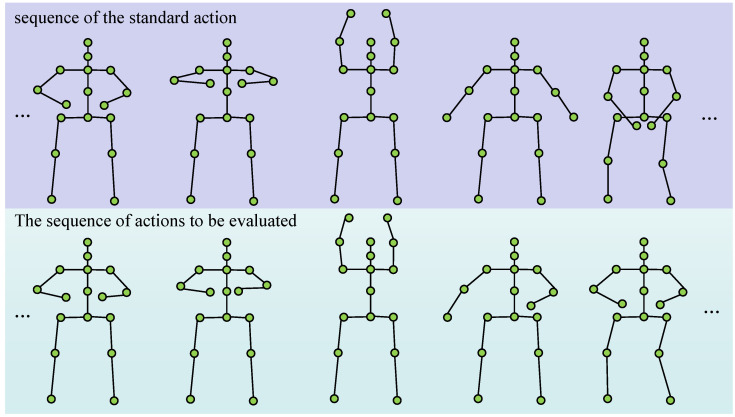
Examples of traditional Chinese Qigong actions. After extracting the skeleton key points through MMPOSE, the motion characteristics of Qigong actions can be clearly represented.

The dataset comprises 5887 video clips, averaging 268 per each of the 22 Qigong sub-actions (from Ba Duan Jin and Yi Jin Jing) shown in Figure 5. Raw videos was captured using standard mobile phone cameras (720p, 30 fps) in unconstrained, real-world environments (e.g., dormitories, outdoors, homes) under varied and uncontrolled lighting conditions. A diverse cohort of 2808 participants (1466 male, 1342 female; aged 18–23) with balanced experience levels introduces significant inter-subject heterogeneity, stemming from wide variations in height (150–185 cm), body proportions, and individual execution styles. This intentional diversity makes the dataset a challenging and realistic benchmark for AQA. 10 Physical education experts rated each assessable action sample based on student performance, providing a reference for evaluating method performance.

## 4. Experiment

### 4.1. Implement Details

All experiments are conducted on a system equipped with an 11th Intel (Ontario, California, USA) Core(TM) i7-11700K @ 3.60 GHz processor, dual NVIDIA (Santa Clara, California, USA) RTX 4090 GPUs (24 GB each), and running Ubuntu 22.04. The primary programming language is Python (version 3.12), with major libraries including PyTorch 1.9.1 and CUML.

This study adopts several commonly used metrics for AQA to evaluate the effectiveness of the proposed method. Most prior studies utilize the Spearman rank correlation coefficient to measure the association between true and predicted quality scores.

(15) $$\begin{array}{cccc} & & \mathrm{Sp}.\mathrm{Corr}=\rho =1-\frac{6\sum d_{i}^{2}}{n(n^{2}-1)} & \end{array}$$

In Equation (15), ρ denotes the Spearman rank correlation coefficient, $d_{i}$ is the difference between the ranks of corresponding values from two samples, and *n* is the number of samples.

Furthermore, to compare the prediction performance of different algorithms further, this study employs Mean Absolute Error (MAE), Relative Mean Absolute Error (RMAE), and Mean Squared Error (MSE) as supplementary assessment metrics. The specific formulas for these metrics are provided in Equations (16)–(18). These metrics facilitate an effective comparison with previous research results.

(16) $$\begin{array}{cccc} & & MAE=\frac{1}{n}\sum_{i=1}^{n}\left|y_{i}-{\hat{y}}_{i}\right| & \end{array}$$

(17) $$\begin{array}{cccc} & & RMAE=\frac{1}{n}\sum_{i=1}^{n}\frac{\left|y_{i}-{\hat{y}}_{i}\right|}{y_{i}} & \end{array}$$

(18) $$\begin{array}{cccc} & & MSE=\frac{1}{n}\sum_{i=1}^{n}{\left(y_{i}-{\hat{y}}_{i}\right)}^{2} & \end{array}$$

Here, $y_{i}$ represents the ground truth score for the *i*-th sample, ${\hat{y}}_{i}$ denotes the predicted score, and *n* is the total number of samples.

To establish a gold standard, 500 samples were evaluated by 10 human experts of comparable expertise. Their ratings served as the ground truth labels for our comparative experiments.

### 4.2. Algorithm Complexity Analysis

The computational efficiency of the QAQA algorithm stems from its hierarchical, coarse-to-fine processing of time series during recursion. This efficiency is governed by two key constraints. First, an anomaly-derived parameter t∈[0,1], dynamically adjusts the vertical and horizontal path penalties within the cost matrix update. Second, the coarse-grained alignment path is projected onto the fine-grained level, constraining the search space to a window whose size is dictated by the hyperparameter radius. The radius parameter directly governs the trade-off between computational complexity and precision: a smaller radius reduces computation by narrowing the search window but risks missing the optimal path, whereas a larger radius increases precision at a higher computational cost.

QAQA further improves efficiency by decomposing the problem into O(logn) levels using a multi-resolution approach. At each level, QAQA does not compute the full n×n cost matrix. Instead, it calculates a narrow band of cells with width 2r, where *r* is a relatively small constant. Given that the sequence length at a given level is $n^{\prime }$, the time complexity at each level is $O(n^{\prime }\;\cdot \;r)$, which is effectively $O(n^{\prime })$. Therefore, given the narrow computation window, the time complexity at each level is O(n).

(19) $$\begin{array}{cccc} & & TimeComplexity=\sum_{k=0}^{\log n}O\left(\frac{n}{2^{k}}\right)=O\left(n\right)\;\cdot \;\sum_{k=0}^{\log n}\frac{1}{2^{k}} & \end{array}$$

Equation (19) illustrates the overall time complexity. Here, *k* denotes the level index, where $k\in [0,\log_{2}n-1]$. The decomposition process resembles binary division, resulting in $\log_{2}n$ levels in total. At each level, the sequence length is halved: n→n/2→n/4→…→ threshold. Consequently, the time complexity at each level can be expressed as $O(\frac{n}{2^{k}})$. The series $\sum_{k=0}^{\log\;n}\frac{1}{2^{k}}$ is geometric and converges to a constant (specifically, less than 2). As a result, the total computational workload remains linear, so the overall time complexity is O(n).

Regarding space complexity, QAQA employs ACDTW [41] for scoring. This approach requires three matrices of size $O(n^{2})$ to record the frequency of matching for each point in the time series. Therefore, the space complexity of the algorithm is $O(n^{2})$.

### 4.3. Comparison Study

As shown in Table 2, both ACDTW and QAQA with the anomaly detection module achieve superior scoring performance, significantly outperforming their counterparts without the module. This shows the positive impact of the anomaly detection module in improving the accuracy of the scoring system.

ACDTW [41], as an unsupervised (indirect) scoring method, inherently provides robust scoring accuracy. Compared to PG-MI, the best-performing regression method, ACDTW with the anomaly detection module achieved a 19.27% improvement in Spearman rank correlation. Similarly, QAQA exhibited the greatest improvement among all methods, increasing Spearman rank correlation by approximately 19.53% compared to supervised methods.

These results suggest that traditional supervised learning methods are often tailored for specific scenarios and actions, and their built-in scoring modules struggle to adapt to the complex movements found in Qigong. In contrast, indirect scoring methods can more accurately capture the unique characteristics of Qigong actions, resulting in more realistic assessments. Moreover, indirect scoring methods demonstrate stronger generalization capability, as they do not require pre-training on labeled scoring data.

As presented in Table 3, a comparative analysis of unsupervised clustering algorithms, including prototype-based (K-Means) and ensemble-based (isolation forest), was conducted. The DBSCAN-based anomaly detection demonstrated superior performance, achieving an improvement of up to 11.76% over other methods. This effectiveness can be attributed to two key aspects: the high consistency between the inherent physical characteristics of acceleration and DBSCAN’s density-based assumption, and its ability to capture acceleration anomalies in euclidean space without a priori knowledge of their shape.

### 4.4. Ablation Study

#### 4.4.1. Parameter Ablation Analysis

In parameter ablation experiments, we compare accuracy and computational time by varying the radius parameter to identify the optimal value for QAQA. The scoring results of the ACDTW on the Chinese traditional Qigong dataset are used as baseline to evaluate the differences among various unsupervised algorithms.

As shown in Table 4, the original DTW algorithm exhibits the most significant error. For QAQA, a smaller radius (e.g., 2) yields higher error, while increasing the radius to 10 achieves the smallest error, and the Spearman rank correlation coefficient for QAQA with a radius of 2 is 0.021 lower than that with a radius of 10. Table 5 presents the average computation times for different algorithms and radius settings. When processing the same dataset, the QAQA scores closely match those of ACDTW, while its computational efficiency is significantly better than that of both DTW and ACDTW.

In general, setting a larger radius in QAQA improves prediction accuracy but also increases time complexity. Conversely, a smaller radius can speed up computation but may reduce precision. When the radius is set to 10, the prediction results are closest to those of the ACDTW algorithm, with a minimum MAE of 0.5595, representing the best prediction performance. Moreover, in terms of time consumption, the average computation time is reduced by 2.96 s compared to QAQA with a larger radius, significantly improving efficiency without compromising accuracy. We found when the radius is set to 2, the results differ substantially, but the computation time decreases to just 0.15 s, making it suitable for large-scale evaluation scenarios. Therefore, in practical applications, different radius values can be selected to balance the trade-off between computational speed and precision.

**Figure 6:**
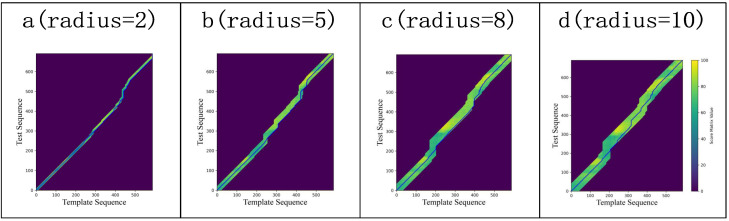
QAQA’s backtracking paths across different radius parameters: The radius range regulates the search path, further determining the final scoring outcomes.

The radius parameter also limits the search range of the backtracking path. To further illustrate the impact of different radius settings, Figure 6 visualizes the shapes of the backtracking path under various radius. In the figure, dark areas represent uncalculated and unupdated cells, while light areas show the frame-by-frame action scores between the template and test sequences, with brighter regions indicating higher similarity.

For instance, in Figure 6a, with a radius of 2, the search range is narrow. In contrast, Figure 6d, with a radius of 10, allows the backtracking path to explore regions with smaller action differences, achieving closer action alignment. However, setting the radius too small may affect the final accuracy, since the optimal backtracking path may lie outside the restricted window. Moreover, reducing the search range also leads to a shorter computational time.

#### 4.4.2. Parameter Sensitivity Analysis

To evaluate the robustness of the proposed anomaly-aware threshold coefficient t in Equation (5), we performed a sensitivity analysis on its binning configuration, with results summarized in Table 6. The proposed setting achieved superior performance with a Sp.Corr of 0.9749 and an MAE of 0.5595. In contrast, widening the bins led to a 0.87% decrease in Sp.Corr and a 9.3% increase in MAE due to reduced penalty granularity. Conversely, narrowing the bins resulted in a 1.04% Sp.Corr drop and an 18.8% MAE increase by over-penalizing minor deviations. The continuous variant was the least effective, confirming that discrete penalty levels are better suited for this task. This analysis validates that our setting provides a robust balance between sensitivity and tolerance.

### 4.5. Case Study of Anomaly Detection Module

**Figure 7:**
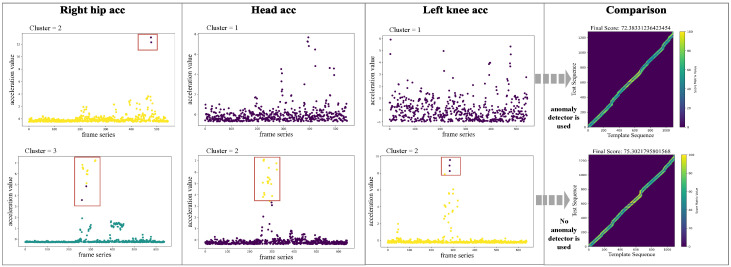
Comparison of acceleration clustering for three keypoints between a standard template (**top**) and a test sample (**bottom**). Different colors correspond to different acceleration clusters. The results reveal execution anomalies, particularly in the head joint of the test sample, which exhibits a significant distributional shift compared to the template.

Abnormal joint data can degrade action assessment accuracy. Figure 7 illustrates this by visualizing clustered keypoint accelerations, comparing a standard template against a test sample with anomalies. The figure reveals that while some joints like the left knee exhibit stable acceleration patterns similar to the template, others, such as the head, show significant distributional divergence and numerous outliers, indicating improper execution. Our anomaly detection module is designed to identify and penalize these deviations. As shown in the final column, activating this module (threshold parameter t = 0.15) dynamically adjusts the score from 75.3 to 72.3. This demonstrates that our mechanism produces more robust and realistic evaluations by systematically accounting for keypoint anomalies.

## 5. Discussion

In this paper, we proposed a fast and robust framework for AQA that addresses key limitations in existing methods for daily exercise analysis. By integrating a novel, DBSCAN-based anomaly detection module with our efficient QAQA scoring algorithm, our approach effectively handles noisy skeletal data and operates without the need for data annotation. The QAQA algorithm’s linear time complexity marks a significant improvement over traditional DTW-based techniques, enabling real-time analysis of long video sequences without compromising accuracy.

Our empirical evaluation confirms the superiority of the proposed framework. On a newly established, large-scale dataset for traditional Chinese Qigong, our method consistently outperformed state-of-the-art approaches in both computational efficiency and assessment accuracy. This dataset itself represents a valuable contribution to the community, providing a robust benchmark for future research in intelligent sports education.

Despite its strong performance, this work opens several avenues for future research. The anomaly detection module could be enhanced by incorporating spatio-temporal graph modeling to capture more complex error patterns. Furthermore, a hierarchical assessment framework could be developed to provide multi-level feedback tailored to actions of varying complexity. These future directions promise to further advance the practical application of AQA in real-world teaching environments and forge new pathways for the intelligent development of traditional sports.

## Figures and Tables

**Table 1:** Human limb Angle feature extraction number.

Angle_id	Key Point A	Key Point B	Key Point C
I	8 Thorax	9 Neck	14 R-shoulder
II	8 Thorax	0 Hip	14 R-shoulder
III	14 R-shoulder	8 Thorax	15 R-elbow
IV	15 R-elbow	14 R-shoulder	16 R-wrist
V	11 L-shoulder	9 Thorax	12 L-elbow
VI	12 L-elbow	11 L-shoulder	13 L-wrist
VII	0 Hip	8 Thorax	1 R-hip
VIII	1 R-hip	0 Hip	2 R-knee
IX	2 R-knee	1 R-hip	3 R-foot
X	4 L-hip	0 Hip	5 L-knee
XI	5 L-knee	4 L-hip	6 L-foot

**Table 2:** Results of comparative experiment. The best results are indicated in **bold**, and the second best ones are underlined. The 95% confidence interval (CI) for Spearman’s rank correlation coefficient is reported to quantify the uncertainty of the estimate, the *p*-values are derived from the paired Wilcoxon signed rank test. ✗: Without anomaly detection; ✓: with anomaly detection; ↑: higher is better.

Method	Anomaly Detection Module	Sp. Corr ↑	95% CI	*p*-Value
ST-GCN [49]	✗	0.4782	[0.4673,0.4891]	$3.2\times 10^{-14}$
MUSDL [50]	✗	0.5153	[0.5101,0.5205]	$7.8\times 10^{-13}$
HGCN [9]	✗	0.5958	[0.5897,0.6019]	$4.1\times 10^{-11}$
CoRe [51]	✗	0.6584	[0.6416,0.6752]	$9.6\times 10^{-10}$
ASGTN [52]	✗	0.6259	[0.6170,0.6348]	$2.3\times 10^{-10}$
TPT [53]	✗	0.7219	[0.7942,0.8066]	$1.6\times 10^{-08}$
PG-MI [54]	✗	0.7845	[0.7518,0.8172]	$9.8\times 10^{-07}$
ACDTW [41]	✗	0.8004	[0.7914,0.8091]	$3.7\times 10^{-06}$
ACDTW [41]	✓	0.9718	[0.9542,0.9894]	$9.4\times 10^{-04}$
our QAQA	✗	0.8110	[0.7867,0.8353]	–
our QAQA	✓	**0.9749**	[0.9681,0.9817]	–

**Table 3:** The impact of different clustering algorithms in the anomaly detection module on the accuracy of AQA, among which the detection based on the DBSCAN algorithm demonstrates the best performance. The best results are indicated in **bold**, ↑: Higher is better, and ↓: lower is better.

Methods	Clustering Algorithm	Sp. Corr ↑	MAE ↓	RMAE ↓
ACDTW	K-Means [55]	0.8154	2.0470	0.0790
ACDTW	Isolation Forest [56]	0.8498	1.7841	0.0344
ACDTW	DBSCAN [48]	0.9718	0.6571	0.0075
our QAQA	K-Means [55]	0.8323	1.8012	0.0319
our QAQA	Isolation Forest [56]	0.9356	0.6724	0.0073
our QAQA	DBSCAN [48]	**0.9749**	**0.5595**	**0.0068**

**Table 4:** Ablation study of the radius parameter (accuracy). The best results are indicated in **bold**, and the second best ones are underlined. ↑: Higher is better, and ↓: lower is better.

Method	Radius	MSE ↓	MAE ↓	RMAE(s) ↓
DTW [34]	–	31.2057	4.5734	0.0571
QAQA	2	7.8390	1.7183	0.0210
	5	4.1399	1.0650	0.0130
	8	2.5128	0.7039	0.0086
	10	**1.9945**	**0.5595**	**0.0068**

**Table 5:** Ablation study of the radius parameter (computation time). The best results are indicated in **bold**, and the second best ones are underlined. ↑: Higher is better, and ↓: lower is better.

Algorithm	Radius	Avg Time ↓ (s)
DTW [34]	–	0.8452
ACDTW [41]	–	3.4620
QAQA	2	**0.1500**
	5	0.3214
	8	0.5020
	10	0.6228

**Table 6:** The result of the parameter sensitivity analysis. ↑: Higher is better, and ↓: lower is better.

Variant	Bin Boundaries	t Values	Sp. Corr ↑	MAE ↓
Original Setting	[0, 0–0.3, 0.3–0.5, >0.5]	$\left[1,0.2,0.15,0.1\right]$	0.9749	0.5595
Wider Bins	[0, 0–0.4, 0.4–0.7, >0.7]	$\left[1,0.25,0.18,0.12\right]$	0.9662	0.6087
Narrower Bins	[0, 0–0.2, 0.2–0.4, >0.4]	$\left[1,0.18,0.12,0.08\right]$	0.9645	0.6053
Continuous	–	1-ratio	0.9331	0.7524

## Data Availability

The datasets used and/or analysed during the current study are available from the corresponding author upon reasonable request only for the purpose of academic research. It is available via Google Drive: https://drive.google.com/drive/folders/1bPnoIrRZLBV8fPS1wo5XqhktYgJVjEy3?usp=sharing (accessed on 11 September 2025). Our academic research-only code is open sourced at https://github.com/waHAHJIAHAO/QAQA-main (accessed on 11 September 2025).

## Abbreviations

The following abbreviations are used in this manuscript:

AQA	Action Quality Assessment
DTW	Dynamic Time Warping
